# Correction to: The role of oral co-trimoxazole in treating Nocardia farcinica keratitis: a case report

**DOI:** 10.1186/s12348-017-0143-2

**Published:** 2018-01-08

**Authors:** Neharika Sharma, Stephen O’Hagan

**Affiliations:** 1 Cairns Base Hospital, 165 The Esplanade, Cairns, QLD Australia; 2 James Cook University, 1 James Cook Drive, Townsville City, QLD Australia

## Correction

This article [1] was unintentionally published twice in this journal, by the same authors.

The following [1] should be considered the version of record and used for citation purposes: “Neharika Sharma and Stephen O’Hagan, The role of oral co-trimoxazole in treating Nocardia farcinica keratitis: a case report. Journal of Ophthalmic Inflammation and Infection (2016) 6:21 DOI 10.1186/s12348-016-0087-y”.

The duplicate [2]: “Neharika Sharma and Stephen O’Hagan, The role of oral co-trimoxazole in treating Nocardia farcinica keratitis: a case report. Journal of Ophthalmic Inflammation and Infection (2016) 6:23 DOI 10.1186/s12348-016-0091-2” is to be ignored.

BioMed Central apologizes to the readers of the journal for not detecting the duplication during the publication process.
